# Perception of tobacco use in young adults in urban India: a qualitative exploration with relevant health policy analysis

**DOI:** 10.3332/ecancer.2019.915

**Published:** 2019-03-28

**Authors:** Soumita Ghose, Alok Sardar, Suman Shiva, Brega Ellen Mullan, Soumitra S Datta

**Affiliations:** 1 Department of Medical Administration, Tata Medical Centre, Kolkata 700156, India; 2 Department of Hospital Management, Techno India, Kolkata 700091 India; 3 Faculty of Economics and Business, University of Groningen, 9712 CP Groningen, The Netherlands; 4 Newcastle University Business School, Newcastle University, Newcastle upon Tyne NE1 7RU, UK; 5 Department of Palliative Care and Psycho-oncology, Tata Medical Centre, Kolkata 700156, India; 6 UCL EGA Institute for Women’s Health, University College London, London WC1E 6BT, UK

**Keywords:** cancer, tobacco, youth, tobacco policy, tobacco law, India, smoking, adolescent

## Abstract

Tobacco is one of the biggest global health concerns of this century with a significant contribution to the increasing burden of cancers, chronic diseases and associated mortality. Tobacco-related cancers are one of the commonest causes of cancer-related mortality in low- and middle-income countries (LMICs). The tobacco epidemic is constantly on the rise, affecting LMICs in particular due to a lack of awareness in the population, insufficient health infrastructure and weak regulatory interventions. India is home to the world’s largest youth population and a large percentage of them take up tobacco at a very young age, leading to subsequent habit formation. There is limited evidence in published research from India on young people’s perceptions on the use and control of tobacco.

This qualitative study has attempted to bridge that knowledge gap; a thematic analysis was used on the qualitative data gathered from young university students who participated in interviews and focus group discussions, which was then compared and contrasted with a critical analysis of India’s national tobacco control measures. It employed a health policy analysis framework to understand how gaps in the national tobacco control initiatives contribute towards tobacco use in young people and what opportunities for policy reform exist.

The main results revealed social and behavioural factors, peer dynamics and lack of awareness to be majorly influencing the tobacco debut and use in youth. Some other important findings emerged such as a lack of available support for tobacco cessation, leading to failure in quitting, a lack of understanding about the ill effects of tobacco and an overall lack of belief in the existing tobacco control measures. The qualitative results were further triangulated by the critical analysis of the national tobacco control policies, comparing them with the WHO Framework Convention on Tobacco Control. Juxtaposition of the qualitative research findings with the policy analysis reveals possible gaps in implementation of the tobacco laws. The findings from this study will inform health policymakers, public health professionals, clinicians, the government and other voluntary organisations to strengthen national tobacco control efforts.

## Background

Tobacco is the single most important preventable cause of death globally [1]. In adults, it is the second leading cause of mortality, translating to more than 6 million people dying yearly due to tobacco-related diseases [2]. Tobacco use is associated with several types of cancers, which are very common in low- and middle-income countries (LMICs), contributing to 50% of all cancers in men and 20% in women [2, 3]. By 2030, tobacco is estimated to kill around 10 million people a year [2–5]. The epicentre of this tobacco epidemic remains in LMICs, with 70% of the estimated deaths and 80% of the total 1 billion smokers in the world coming from there [4–8]. The most commonly used tobacco products are cigarettes, containing many chemicals with proven carcinogenic properties, pesticides and tar [9, 10]. Other than cigarettes, there are many other smoked, non-smoked, chewable, drinkable forms of tobacco that are often mistakenly assumed to be non-hazardous by users [11]. Contrary to popular belief, scientific studies show that all forms of tobacco use can be lethal [12].

India is home to 275 million tobacco users and is the second largest producer and user of tobacco products after China [13]. The country is also known for widespread production and consumption of many smokeless forms of tobacco. In India, one-third of the population uses tobacco in some form [7] and it kills over 1 million people per year [14]. Due to the massive use of various smokeless forms of tobacco, often homemade, India has one of the highest rates of oral cancer in the world with an annual incidence as high as 10 per 100,000 among males [15–17].

Prevention of early tobacco debut and subsequent use of tobacco in youth is one of the critical ways of reducing the burden of non-communicable diseases, including cancers in the world. India is currently home to the world’s largest youth population [18]. Research indicates that addiction to tobacco is initiated in adolescence and youth in a majority of cases [19–21]. Many of these users demonstrate a very early smoking debut in the age group of 15–24 years [19–21] and a significant proportion of India’s current population fall into this group [20]. In addition, a majority of the tobacco-related deaths happen at a very young age [3, 6]. Tobacco companies target the youth aggressively with special products such as flavoured and specially packaged cigarettes; several points of sale advertising is also widely used in the country, such as displays in shops and supermarkets, on social media and celebrity endorsements which have been proven to increase the youth’s susceptibility towards tobacco debut [22, 23]. Therefore, focusing tobacco control measures towards the youth will have far-reaching effects in changing the population’s behaviour and lifestyle in the long term. The Global Youth Tobacco Survey indicates that 15.5% students are likely to take up tobacco in India annually [24], but there is limited information on how today’s youth perceive smoking and using other tobacco products, why they use tobacco as a habit-forming agent and most importantly, what they think of the anti-tobacco campaigns and legislative control measures that exist today in the country. This understanding of their perceptions is critical in identifying gaps in their awareness and in the effective implementation of tobacco control laws of the land.

The WHO Framework Convention on Tobacco Control (FCTC) was the first international treaty on tobacco control that was accepted by the World Health Assembly in 2003. In India, following input from national public health experts, the government passed a legislation called ‘Cigarettes and Other Tobacco Products Act (COTPA)’, which is the main legal framework that regulates production, distribution, sales and use of tobacco products in the country. Even after 12 years of signing the treaty, the number of published policy analysis addressing tobacco control in India is scarce even though there is a significant amount of analysis done in other countries like China and Brazil. This makes the need for a policy analysis stronger.

This paper is divided into two sections; the first section focuses on the results of a qualitative research study exploring the knowledge gaps around smoking debut and tobacco use in urban youth. This qualitative arm of the study also looked into the perception of young people against the existing anti-tobacco campaigns. The second section of the paper analyses and evaluates the existing legislation on tobacco control in India using a pre-validated analysis framework and compares the findings of the policy analysis with the results from the qualitative study. Finally, the paper critically discusses the relevant gaps and limitations of the national tobacco control initiatives in addressing the tobacco epidemic in the urban youth in India.

This is the first paper from India to combine tobacco use in youth using qualitative methods and comparing it with a critical analysis of tobacco-related policies of a national and international treaty. This method of exploration of an individual’s behaviour in the context of the national and international policy makes the findings of the study relevant for future policy development and implementation in reducing the burden of several preventable tobacco-related cancers.

## Methodology

### Study design

The study used a qualitative design to explore the perception of tobacco use in youth, in particular, the enablers of tobacco debut and subsequent use and the barriers to successful implementation of tobacco control measures, all of this examined in light of the policy framework analysis of tobacco control related legislations. Considering the objective of the study, which is to explore how the youth perceive tobacco use and the tobacco control measures of the country, a qualitative study design was followed. This approach helped to ensure the richness of data, as free and unbiased communication was encouraged to understand the phenomena. The qualitative design also ensured understanding the youth from their frame of reference and their experience of the phenomena of tobacco debut and use. In addition, a pre-validated policy analysis framework was followed in evaluating the national tobacco control policies and other international conventions that have a major bearing on India. The study uses a novel methodology of juxtaposing the qualitative research findings side by side with the results of a health policy analysis.

### Setting

The qualitative study was conducted in students enrolled in a university located in the metropolitan city of Kolkata that admits students from different states of India, such as West Bengal, Jharkhand, Bihar, Uttar Pradesh and others. A significant number of these students get financial support in terms of scholarship from the institution being supportive of students coming from economically deprived backgrounds. This ensured the recruitment of participants from a variety of socio-economic backgrounds. Eighty percent of global tobacco use are in LMICs; and in India, 42.4% of men, 14.2% of women and 28.6% (266.8 million) of all adults currently use tobacco according to the Global Adult Tobacco Survey-2 published in 2017 [24]. This dictates the urgency of exploring the tobacco epidemic in India.

### Eligibility criteria for the study

The eligibility criteria to participate in the study were fairly broad as described below:

*Inclusion criteria:* Young adults more than 18 years of age, studying in the university were eligible to participate in the study.

*Exclusion criteria:* Since this was an interview-based study on a non-clinical healthy population, there were no specific exclusion criteria. The research team did not approach students under 18 years of age as the researchers could only recruit adult participants according to the study design and ethical approval.

### Research team

The research team consisted of a public health researcher with expertise in qualitative methods, a psychiatrist practicing and researching full-time in the field of psycho-oncology, a faculty of health management and two visiting interns with a background in health economics and business, respectively, who were also students themselves during the time of the study. This diverse skill set of the research team was immensely helpful in the qualitative data generation and exploring the data during analysis. Having external peer interviewers who did not have any prior contacts with the study participants before the commencement of the study is in line with the Consolidated Criteria for Reporting Qualitative Research (COREC) guidelines for qualitative research [25].

### Recruitment of participants

The study was conducted inside the campus of the university after obtaining the necessary approvals. The students were informed about the study by their faculty and also by identified peer leaders, and were invited to voluntarily participate in the study. The research team members visited the university for data collection. The university provided a space for the interviews to happen in private; however, some students preferred to be interviewed in more informal spaces such as the university’s opens spaces—this was allowed.

### Sampling method

A purposeful sampling was employed by the researchers as advocated by Collins [26] and included a diverse range of students. In purposeful sampling, the researchers recruit respondents based on diversity as defined by a pre-determined sampling boundary often based on age, gender, domicile and other specific parameters carefully selected so as to generate adequate and sufficient data so that generalisation and theory development is possible [27]. As opposed to quantitative research, where probabilistic random sampling is used to improve external generalisation and offer equal chances to every member of the population of interest of being selected, in qualitative research, the researcher aspires to generate new theories by obtaining new insights or fresh perspective on the phenomenon of interest combining common and rare points of views [27]. Thus, qualitative researchers often prefer to recruit respondents who are articulate about their views that yield a depth of understanding and unique perspective to the research question that is being explored [26]. By nature, qualitative research is exploratory. In this particular research, personal tobacco use of the students was not taken as a deciding factor for recruitment as the researchers were interested in the views of users and also those young people who were not users of tobacco. The sample size of participants was determined by the principles of data saturation as advocated in gold standard qualitative research [28]. Guest *et al* [28] defined data saturation as ‘the point in data collection and analysis at which new interviews/information give rise to little or no new codes’.

### Explaining the study and obtaining informed consent

All students who approached the researchers were briefly informed about the study. While explaining the study, the researchers were mindful that they should be brief and not discuss the conceptual basis of the study at length so that they did not unconsciously influence the opinion of the students. The potential participants were told that their participation in the study was entirely voluntary and that they could withdraw their consent any point. They were told that the researcher may follow some pre-determined interview stems while asking about tobacco use, but the participants are free to talk about anything they felt relevant to the topic of the research. The respondents were informed that for the purpose of the rigour of the research, their interviews would be audio-recorded but their responses would be kept confidential and none of the personal identifiers will be ever made public. Having explained the process, if they were keen to participate, they were recruited after they provided written informed consent. A similar brief was also given during the two focus group discussions and consenting was carried out.

### Data collection methods for the qualitative study

The researchers conducted in-depth interviews, focus group discussions and participant observations. Basic socio-demographic data were collected in order to describe the sample recruited for the study. Interviews and focus groups were audio-recorded and later transcribed verbatim.

### In-depth interviews

Trained young researchers conducted the in-depth interviews on the university campus; this ensured free and uninhibited communication between the respondents and interviewers. Participants were given the choice of a private office or a more informal setting (such as the college grounds) as deemed comfortable by the interviewee. The researchers followed a semi-structured prompt/stem for the interviews but the discussion was free-flowing and not restricted to these stems. As the research progressed, additional areas that came to light in previous interviews were explored further as advocated in qualitative research. The open-ended interview allowed the young people to speak about topics that they considered important with respect to their tobacco habits.

**Interview Stems used in the study:**

- What do you think about tobacco use amongst your peers and friends in general?
- When were you first offered a smoke or any other form of tobacco?
- Do you and your friends smoke?
- Would you describe your first experience of use?
- When was the second experience and subsequent use?
- What are the ill-effects or risks of tobacco use?
- What are the benefits of tobacco use (if any)?
- What prevents people from taking up smoking?
- Why do young people continue smoking in spite of knowing about the harm?
- Do you know anyone who has quit smoking? What helped them?
- What do you think about anti-tobacco campaigns in India?
- Do you the think anti-tobacco measures work?
- What are the other ways you think tobacco use can be reduced?

### Focus group interviews

Researchers moderated the focused groups using structured guidelines. The focus group interviews explored with more open-ended questions. The focus group discussions centred around the same questions as in the interviews, in addition, further discussions centred on the government’s initiatives to control tobacco use and its effectiveness, reasons for and against smoking, feasibility of banning tobacco and other related topics as brought up by the interviewees.

### Participant observation

Two participant observations were conducted in the same university campus. All observations were kept anonymous and confidential. The participant observations were done in the food kiosks located right outside the main gate of the campus where students come for eating; these food kiosks also sell tobacco products to the students.

### Ethics

The study was cleared by the Institutional Ethics Committee of the Tata Medical Centre, Kolkata, India [EC/TMC/96/17] and adhered to all national ethical guidelines.

### Data collection for policy analysis

The policy analysis section is based on publicly available secondary data covering the time frame from 2004 to present. Apart from the main WHO FCTC treaty and India’s tobacco control law (COTPA); a database search was conducted for published academic sources. A licensed search platform was used and all relevant research and opinion pieces commenting on the tobacco policy/regulation in India that could be collected were examined using the framework of policy analysis.

## Data analysis

### Qualitative data analysis

In-depth interviews and focus group discussions were transcribed verbatim. All data from interviews and focus groups were organised using NVivo version 9.0. Data collection and data analysis were conducted simultaneously. All interviews were coded by two independent researchers. Data analysis was conducted following principles of thematic analysis as suggested by Braun and Clark [29]. The inductive coding consisted of reading all transcripts and participant observation notes. Patterns from the data were generated and then organised as per similar attribute and used for creating themes. Similar themes were then compared and contrasted to create global themes.

### Policy analysis

Public policy analysis is defined as the study of how, why and to what effect governments pursue particular courses of action and inaction [30]. Health policy contexts are often highly political and debated. Health policy analysis is thus a social, as well as a political activity [31]. According to the trajectory of modern health policy analysis, policy reform is a political process, which needs to take into consideration the reasons why policy outcomes failed to emerge originally. This paper bases its foundation on this new paradigm of policy thinking as discussed in the works of Walt and Gilson [32] which analyses the policy world in its entirety in the form of a ‘thick description’ [32, 33] other than only its content matter. This considers the complexity with which public health policies are set in today’s world, the content rarely reaches consensus and is often faced with multiple conflicts and resistance. A policy triangle framework of analysis developed by Walt and Gilson [32] is followed in this paper. This was specifically developed for the health sector and found its use in a wide array of health issues in different countries, especially LMICs. Grounded in political economic perspective, this framework critically reviews a policy and attempts to look at ‘what explains what happened’ rather than only looking at ‘what happened’ [33]. The policy arm of the paper critically analyses relevant sections of India’s tobacco control policies—taking its lead from the qualitative arm, comparing them with the WHO FCTC treaty, identifying barriers and gaps to understand how they are impacting the use of tobacco in young people, and recommending opportunities for policy reforms. The current research juxtaposed and critically examined the qualitative findings of the tobacco use in young people alongside the findings of the policy analysis.

## Results

### Participant characteristics

A total of 30 participants took part in the in-depth interviewing and focus group discussions. The baseline demographic characteristics of the study respondents are presented in Table 1. The youngest participant was 18 years old and the oldest was 22 years of age; one-third of the respondents were women. A majority (70%) of the youth who participated in the study had one or two members of their immediate family who were smokers. Around half (53%) of the participants said that their fathers are current smokers and all of them said that either some of their friends or all of their friends are smokers. Among the respondents, 75% of all males were smokers/users of other forms and 50% among all females said they smoke cigarettes.

### Results of the qualitative analysis

Thematic analysis of the qualitative data generated four global themes, namely, *why people start smoking? Knowledge of ill effects of tobacco*, *perceptions about tobacco use and quitting* and *views on tobacco control measures of the country.*

## Why do people start smoking?

Smoking was largely considered a social behaviour by the young adults in this study and the factors that influenced smoking initiation were (a) Peer influence and social desirability (b) Curiosity about experimenting with smoking (c) Identifying smoking as a method of stress relief. These factors were reported as influencers by the youth who are smokers, as well as the ones who said that they do not smoke.

### Peer influence and social desirability

Many of the participants pointed out the role of peers in tobacco initiation in youth and encouraging young people to smoke their first cigarette. Social desirability to feel more inclusive in a group and smoking to look smart enough to belong to that group was reported as a major factor in smoking initiation.

We felt that if we had it (cigarettes), we can be part of that group… (male, 18, smokes occasionally)

Most of the young adults reported that they first started smoking with their friends or senior peers who had offered a cigarette to be smoked in ‘counter’ which colloquially means sharing.

To be honest, the first time I smoked, it was in front of a girl. She was a chain smoker and she was taunting me that I was a kid and not supposed to smoke and that I was coughing (from the smoke). So, I felt humiliated. At that point of time, I thought that it’s not that difficult. I tried one at first and then after a few more cigarettes I felt ‘normal’ with it (male, 19, smokes)

## Curiosity about experimenting with tobacco

Some of the students reported that they tried their first cigarette to understand why their father smokes regularly. Among the other reasons was common curiosity about tobacco, trying a cigarette as an experimental thing, which later formed a habit, trying the cigarette as it was fun to try something new. Most of these students who had tried cigarettes feeling curious ended up forming a habit; with the exception of only few who didn’t like the taste or the experience and therefore never smoked again.

This might sound a little stupid, but I wanted to know why my father smoked. That has made me start it (male, 19, smokes sometimes)

I want to experience that thing for experimenting (female, 18, smokes often)

### Stress and pressure relief

Stress and subsequent relief from stress after smoking was identified as an influencer. Stress was reported to be of facing examinations, family problems; in addition, associated symptoms of stress as headache, sleeplessness, feeling depressed, etc. were also reported to be controlled by smoking. Many of the young adults reported to be resorting to smoking as a relief from their stress, depression and tensions of personal lives.

I smoke because I get relieved after sitting in class for so many hours and then after going for the coaching centre (male, 18, smokes)

One of my friends actually went through a depression, so started smoking everyday (male, 18, tried it but doesn’t smoke now)

### Knowledge of ill effects of tobacco

Overall, most of the respondents had knowledge about cancer being caused from tobacco and smoking. While many students couldn’t tell anything beyond tobacco causing cancer, a very few had detailed knowledge about other ill effects such as chronic diseases, passive smoking, etc. Two distinct themes came out where one group strongly believed the ill effects to be true regardless of whether they smoked or not; the others were not sure of the ill effects.

### It’s true that smoking causes cancer

This group of respondents could identify and believe cancer (in most of the cases) and lung diseases, asthma, other high-risk behaviour and addictions (in a few of the cases) as the ill effects of tobacco. A number of factors contributed to their knowledge of harmful effects, such as self-education from the Internet, knowledge from a family member who’s a smoker and history of family illness due to smoking.

Yes, I do believe in it and that’s why I try to stop my father from smoking continuously…. I know it causes cancer because I have read many times, and I have seen it in movies ….new channels and all. (Male, 19, smokes)

### It’s not true (unless you smoke a lot), haven’t seen anyone getting cancer from smoking

There were a large proportion of students who did not believe that tobacco causes cancer or other diseases. Furthermore, many of these non-believers also thought that tobacco, if consumed in small quantities, isn’t harmful at all for health.

Yeah, there are negative effects, but I don’t smoke regularly… (female, 20, smokes sometimes)

The primary source of such belief was the fact that they hadn’t seen anyone to suffer from cancer, who had been a smoker.

I don’t know about cancer because I never came across a person who is having too much tobacco and has cancer but I do know it can cause a lung disease (male, 18, smokes once in a while)

One student believed that healthy diet of having fruits could counter the negative effects of smoking.

I try to have a glass of fruit juice daily because that helps with clearing the throat…I eat fruits and healthy food…I can’t get over this smoking thing (breathless for a few seconds)…I have some friends who are doctors and they say you have to puff 30 cigarettes daily for 30 years and then you get this (cancer) (male, 22, smoker)

### Perceptions and concepts on tobacco addiction and quitting

The perception of the young adults on tobacco addiction and how they judged themselves in terms of being tobacco users revealed very diverse viewpoints. It included non-smokers having an opinion that ‘Tobacco is bad for health, I don’t like it and I don’t smoke’, smokers believing ‘I am not an addict’, and few acknowledging that ‘I am an addict and I am trying to quit’ or ‘It is possible to quit’; a few also expressed that ‘It is impossible to quit’. Table 2 captures the spectrum of responses that were given by various participants.

### Views on the tobacco control measures of the country

Most of the study participants believed that the current tobacco control measures such as package warning, movie warnings and pricing and taxes are not effective in stopping tobacco use. With respect to these measures, four specific patterns emerged as discussed below.

### Access is easy to tobacco

The students taking part in this study reported that they had never faced any issue with respect to accessing cigarettes. While most of them started smoking prior to the age of 18, they could easily access cigarettes from shops located around their schools and colleges or from their family members.

For the youth, it is very accessible, you find stalls around the university, it is their profit so they will sell it to you (20, male, non-smoker)

Yes it is like 2 minutes’ walk from me, and before I would just steal it from my father (male, 19, smokes)

### Nothing works; smokers will always find a way

One of the crucial findings of this research was around the perception of youth about ‘package warnings’ and warnings shown in movies. A majority of students in this study strongly felt that the pictorial warnings may be scary and disgusting to look at, but they have no effect whatsoever on the smokers.

Every day I see a person who takes like 4 or 5 packets of cigarettes a day… and I don’t think he even sees that thing (pictorial warning)…. (male, 19, smokes sometimes)

This (movie warning) is not effective, when we are watching a movie we just care about the movie, what the hero will or won’t do, we are not interested, even I sometimes skip the ads (19, female, doesn’t smoke)

The role of increased taxation and pricing came up in the discussions as well.

People would still buy it, if they are not poor they would still buy it…personally for me it (taxation) won’t (stop using) (20, male, smokes)

If the government increases the price by 1–2 rupees, people can still afford it, that wouldn’t make any difference but if the price was increased by a huge amount 10–15 rupees per cigarette, I think people will consume less (22, male, smokes less now)

### It works to some extent but more needs to be done

The students, who believed that the current measures work only to some extent, discussed many things about the possible tobacco control strategies. A suggestion was to integrate tobacco education in schools.

Here most of the students are addicted to smoking, so if you make a campaign in a school it may affect them more than an advertisement (male, 18, smokes rarely)

The group felt that in colleges, there should be personal contact between experts and the students to make the communication more persuasive

I feel if an experienced person went to the places where people smoke and interacted with them to help them understand that this is not making any changes in their life and that it is only affecting their health (male, 18, smokes rarely).

Completely banning tobacco came out to be a strong suggestion from many but some of the students also pointed out that people might resort to other forms of addiction if the banning is not done properly with adequate cessation help.

It (complete ban) would cause a lot of problem (initially), but it could be beneficial for the country (in the longer run) and the new generation (20, male, smokes)

## Results from policy analysis of the national tobacco control law and the national tobacco control programme in India with special reference to tobacco use in young adults

### The global tobacco epidemic and WHO framework convention on tobacco control

Globally, the widespread use of tobacco is one of the biggest public health challenges of this century. It has been recognised as a deterrent to the success of the United Nations Millennium Development Goals [34]. The physical effects of tobacco use do not become evident for years and sometimes decades, leading to the epidemic’s continued rise globally. India is a signatory and a crucial stakeholder to the WHO FCTC, the biggest global initiative in the history of tobacco control [35]. India is home to approximately 275 million tobacco users being the second largest consumer of tobacco in the world [36]. India’s tobacco problem is complex and unique due to the extensive production and use of many smokeless forms of tobacco.

### The tobacco control policies of India

The COTPA passed in 2003 is the single main legislation controlling tobacco products in India till date. Key provisions of COTPA include ban of smoking in public places, prohibition on advertisement and promotion, sale to minors, health warning on packaging and testing of tar and nicotine content, etc. [37]. In addition to COTPA, the Ministry of Health and Family Welfare launched the National Tobacco Control Programme in 2007 to facilitate compliance to FCTC [37]. Indian Government’s reported expenditure on tobacco control was INR 352,000,000 (US$ 5.2 million) [38, 39]. However, the National Tobacco Control Programme remains limited in terms of its coverage [37].

### Protection from exposure to tobacco smoke

The Framework Convention Article 8 recommends providing protection against exposure to second-hand smoke in public places [35]. In India, smoking is completely banned in most public places, such as workplace, hospitals, educational institutions, trains, etc. [40]. However, the law permits a smoking room in airports, hotels and restaurants housing more than 30 people. The penalty for smoking in a public place is INR 200–500 (US$. 3–7) only. This amount is highly affordable and smoking in crowded public places, taxis and open spaces is common. Also, a significant gap remains, as a majority still gets to use a wide variety of chewable tobacco in public and spit on roads and indoors [41]. The smoking ban does not cover 100% of all open spaces, which is the biggest gap between the treaty expectation and the law. Overall, the current law misses out India’s unique use of smokeless tobacco and bans only smoking in public. In the participant observation conducted in our study, students were found to be smoking right outside the college campus and all the food stalls located outside were found to be selling cigarettes and other tobacco products. This verifies the finding that smoking in public places is not strictly banned and tobacco is freely being sold within the vicinity of educational institutions. Due to its unique burden of smokeless tobacco, Indian law needs to target control of its use in public. Instead of declaring public places smoke free, the law needs to emphasise the achievement of tobacco-free public places. Even the WHO FCTC acknowledges this gap and makes smokeless tobacco a significant part of their 2025 goals [42]. Enacting a complete ban on all forms of tobacco use in public places along with an adequate penalty needs to be imposed in order to protect exposure to passive smoking and reduction in overall use of tobacco in public places.

### Packaging and labelling of tobacco products

The FCTC treaty’s article 11 regulates tobacco product packaging and labelling to warn consumers about the ill effects of tobacco [35]. As per a recent amendment, the Indian government mandates health warning labels both in pictures and written to cover 85% of the product packaging on both sides. This amendment puts India among one of the global leaders in health warnings on tobacco [43]. The government also bans the use of misleading information on packaging, such as ‘light’, ‘ultra-light’, ‘low tar’, etc. But implementation remains weak due to certain gaps. Firstly, because of the availability of unpackaged cigarettes, a large proportion of users do not often come across these warnings. Secondly, the packaging of smokeless tobacco commonly used in rural India does not always bear pictorial warnings. And finally, there are cigarettes available in the market with names like ‘light’, ‘smooth’, ‘mild’ and interpretation of the warning images by the illiterate population remains questionable [44]. The majority of students in our study felt that the package labelling and movie warnings are not being effective in controlling the use of tobacco products in addicts. A few of the respondents also pointed out that they prefer to buy loose cigarettes so that they can avoid the pictures on the packets.

### Advertising, promotion and sponsorship of tobacco products

Article 13 of the FCTC requires implementation of a comprehensive ban on tobacco advertising, promotion and sponsorships [35]. The Indian law enforces a ban on direct advertising through most forms of mass media [40]. But it does not comply with the treaty entirely as it allows point of sale advertisement in shops and does not completely ban sponsorships by tobacco companies. The amended Indian rules regulate depiction of tobacco use in the media in collaboration with the Central Board of Film Certification [38, 39]. However, tobacco companies continue to use legal loopholes in the form of surrogate advertisement and brand diversification. Tobacco companies continue to sponsor cultural events, newspapers and magazines, popular shows and hold a prominent place in the Indian advertising world [45]. Most of the respondents in our study reported that they ignore on-screen warnings about the ill effects of tobacco during the portrayal of smoking at the cinema. India experienced a rise in showing on-screen smoking when measures were implemented to control other advertising mediums for tobacco companies [6]; a WHO bulletin on this issue emphasises the need to control the portrayal of such smoking on screen in movies and other programmes. There is evidence from the developed world that a significant percentage of youth take up smoking due to their exposure to on-screen portrayal of tobacco use [6, 8, 16].

### Prohibition of sale to and by minors

Article 16 of the Framework Convention treaty prohibits the sale of tobacco products to and by minors [35]. India faces a huge conflict in complying with this treaty requirement. Currently, the tobacco law prohibits sales to individuals under the age of 18. It also bans sale within 100 yards of educational institutions and sale through any vending machines [40]. It also prohibits the display of tobacco products at points of sale to restrict easy access to minors and bans minors from handling or selling tobacco products. However, compliance remains a major challenge due to the huge amount of child labour being engaged in tobacco manufacturing, widespread sale of loose cigarettes and bidis attracting young users and displayed kiosks of tobacco inside shopping malls, supermarkets and restaurants. The Global youth tobacco survey for India indicates that the proportion of minors purchasing tobacco remains unchanged [46], 56% of minors are able to buy tobacco without being refused sale due to their age. In other studies done by the WHO, it has been reported that laws restricting access to tobacco products have been failing as young people are reported to have easy access to tobacco [22]. The participant observation has clearly revealed that access to cigarettes is not controlled in colleges and age is not verified before selling tobacco products to students. The respondents who reported that they have very easy access to tobacco and cigarettes regardless of their age also reaffirm the same finding.

### Other relevant findings

There are other gaps in the law that make India noncompliant with the Framework Convention treaty. There is no direct provision in the law to promote public awareness on tobacco [35]. Under the National Tobacco Control Programme, US$ 5 million is allocated every year to implementing public awareness, mostly delivered through mass media [46]. But the coverage and effectiveness of such campaigns in rural India is not known. The Global Youth Tobacco Survey suggests that counselling children and adolescents on the effects of tobacco and smoking and emphasising tobacco education as part of the college curriculum are impactful steps that need to be taken on a global scale [47]. Peer influence came out as a major factor for the youth to start smoking. If this impact can be reversed into peers who would act as anti-tobacco champions in schools, the same peer pressure can probably be used as a protective factor. Tobacco education needs to be integrated from school levels, which are the foundation years for forming negative or positive opinions. We have juxtaposed the qualitative findings with the relevant portions of the findings form our policy analysis (Table 3) so that it gives the reader a quick glance at the strengths and gaps of the policy implementation.

Furthermore, the country currently lacks capacity to provide tobacco cessation services. There are only a few cessation centres providing limited coverage and suffering from high loss of follow-ups [37]. Nicotine replacement items and cessation drugs are available but they are not covered under the national health insurance [38, 39]. Progress on this keeps declining and only 9.2% of smokers and 7.6% of smokeless users can recall having received cessation advice from a study [46]. Studies from WHO have revealed cessation pharmacotherapy support as low as 4% and counselling as low as 9.2% offered by healthcare providers in India [48]. The overall knowledge about tobacco and its ill effects in healthcare students and health professionals have also been found to be low [48]. This translates to the lack of counselling and cessation support being offered to young people in the country. In our study, none except one respondent knew about tobacco cessation clinics. In addition, none of the students reported that they had received any counselling or support to quit smoking. This verifies the complete lack of cessation support in communities. For an effective and sustainable intervention, tobacco control needs to be integrated in all health and development agendas of the government. Integration of tobacco control strategies, especially awareness and cessation support with primary and secondary level healthcare delivery is critical to the success of COTPA and the National Tobacco Control Programme [49]. Integration of tobacco control with primary healthcare will not only ensure increased coverage of the rural, uneducated and low socio-economic classes but also better utilise available resources.

### Discussion

It is estimated that approximately 55,500 children and adolescents start their use of tobacco every day in India [8, 16]. This early debut as experimentation with tobacco and cigarettes often leads to nicotine dependence, adverse health consequences and a large majority of tobacco use continues well into adulthood [21]. Compared to the trends of the 1990s, there is a notable increase in the age-specific prevalence of smoking in men aged 15–29 years in 2010 [49]. The results of the qualitative analysis reveals few critical factors in understanding the culture of tobacco use in youth: influencers for tobacco debut, the reasons for continuing to smoke, the inability of the youth to quit smoking even when the wish exists, the knowledge and beliefs systems around the ill effects of tobacco, and the attitude and reaction towards the tobacco control measures that are employed in the country today. The awareness of young people on the harmful effects of tobacco such as cancer was reported in similar studies conducted in developing countries such as Canada [50], and also the factors that influenced the youth regarding tobacco-related behaviour were comparable to a great extent to our findings from India. The youth in Canada reported that they were influenced by peer dynamics and social factors [50] as in our study. When compared to other developing countries, the deterrents of tobacco use and thoughts of quitting, such as worry about family and the perceived health hazards of tobacco use were also reported by studies conducted in Africa [51]. In India, a quantitative exploration revealed social factors, peer influence, social desirability to influence tobacco habits and knowledge of harmful effects to be significantly poor [7]. In our study, certain behavioural aspects of tobacco use in young people have been clearly portrayed in terms of the effects of peer pressure, social desirability, social contrast about smoking being a depiction of freedom and negative family experience about smoking acting as a protective factor against it. A critical theme that emerged in our study was the pattern of continued tobacco use and the inability to quit stemming from a lack of knowledge on how to quit, the constant peer pressure and use of tobacco by peers acting as a deterrent to quitting and the unavailability of an evidence-based support system to assist quitting. The study also pointed out how the anti-tobacco messages play in the mind of young people. While many of them do not completely believe the harmful effects, as they don’t have personal experience, the warnings on mass media and cigarette packets also turn out to be non-impactful for them. When compared with the subgroup of youth who had a personal experience of a family member facing harmful effects of tobacco with those without, the beliefs, awareness and sensitivity towards the ill effects of tobacco use was higher for the affected group. We found some of the young people were also not very convinced regarding the harmful effects of tobacco. The qualitative findings of our study triangulate very well with the findings from the policy analysis. The juxtaposition of the policy gaps put against the study results reveal significant opportunities for improvement in implementing the COTPA and adhering to the WHO FCTC treaty to both of their full effects. The recommendations made in this paper can be more feasibly implemented through appropriate amendments of the existing laws, by adding regulations surrounding them, implementing the changes and generating a people’s voice. We hope a couple of important factors will potentially influence the tobacco control efforts. The second Global Adult Tobacco Survey results highlight an overall increase in cigarette consumption and very low rates of smoking cessation in India [49]. The global health community and the Indian government will likely feel it compelling to make policy revisions to strengthen tobacco control efforts in the country. To this effect, an amendment to the main tobacco control law seems highly feasible. Secondly, with the new Sustainable Development Goals targeting compliance to the FCTC treaty as a priority [52], tobacco laws will play a crucial role in the success of all nations. However, historically such strict enactments of laws have faced resistance from the tobacco industry and the pro-tobacco lobby. The government needs to demonstrate very strong political will to be able to enact these modifications and make tobacco control more efficient in the country. Leaving out tobacco control from the Millennium Development Goals gave the tobacco industry a significant advantage over the last decade. But with emphasis on tobacco in the United Nations’ Sustainable Development Goals, the focus of the global health community is back on the tobacco epidemic. The Sustainable Development Goal 3 targets the strengthening of the implementation of WHO FCTC in all countries [52]; this paper is one of the very few published policy analyses conducted on India’s tobacco law comparing it with a global guideline like the FCTC treaty and triangulating the same with the data on how it is impacting the youth of the country. With papers similar to this, tobacco can be again brought back into the forefront of the government’s public health agenda.

## Strengths of the paper

The current paper analyses the tobacco picture from all aspects, behavioural, social, political and legislative and provides a thorough understanding of the uniqueness of tobacco in India. The paper also provides solutions that are fit for addressing the unique barriers faced in this country.

## Limitations of the paper

This paper has a few important limitations. The analysis relies on the Global Adult Tobacco Survey (GATS) data, results from the qualitative study and other published studies on tobacco use in India. The evidence from published reports may differ from the actual on-ground compliance scenario in different states in India, as the policy arm of this study did not involve any primary collection of data; hence, all the arguments could not be confirmed with key respondents or officials working in the government on tobacco control. In addition, the qualitative arm of the study was conducted in a university situated in a metropolitan city in India; hence, most of the students interviewed are predominantly from urban communities; hence, the perception of tobacco use in rural youth is not adequately addressed by this study. Lastly, there are other widespread environmental and economic concerns due to tobacco farming and manufacturing, which have not been discussed in this paper due to its limited scope.

## Conclusion

The tobacco epidemic continues to rise in the developing world along with the burden from tobacco-associated cancers. By 2020, India is estimated to have the highest rise in tobacco consumption compared to all other countries and deaths from tobacco are estimated to exceed 1.5 million annually [7, 17]. This will pose a significant threat not only to the population health’s but also on the healthcare infrastructure of the country, which is still in the development stage in primary and secondary care with limited infrastructure to screen, diagnose and treat cancers. The tertiary care systems are constantly being overloaded with cancer being diagnosed at advanced stages, causing a significant drain on the finances of patients, healthcare providers and the government’s economic system. It is not feasible to reverse the effects of the tobacco epidemic without bringing tobacco to the top of the public health agenda of the country and without a focus on the prevention of tobacco debut. India has been at the forefront of tobacco control efforts for many decades. When compared to a global standard, the Indian law reveals significant opportunities for improvement and policy reform. This analysis highlights the need for revisiting the tobacco control laws in India, addressing the existing barriers to have significant impacts on the tobacco picture of this country. The results from this study will inform policy from many aspects—the public health experts working on behavioural aspects of tobacco use and effects on tobacco awareness in young people, the government and private sector policymakers on the existing gaps in cessation support and the government responsible for implementation of the tobacco control laws of the land. Long-term engagement with a broad range of stakeholders would be required for tackling the tobacco crisis. Overall, tobacco control needs to be seen not only as a public health issue but also as a fundamental human right.

## Compliance with ethical standards

The authors do not have any conflicts of interest to declare and the study adhered to national ethical guidelines for clinical research.

## Funding statement

Two of the researchers, Suman Shiva and Brega Ellen Mullan, were supported by the Global Experience Opportunity programme run by the University of Newcastle, United Kingdom. Soumita Ghose and Soumitra Shankar Datta were funded and supported for their time by their employer Tata Medical Centre, Kolkata and Alok Sardar was funded and supported for his time by Techno India College, Kolkata.﻿

## Figures and Tables

**Table 1.** Demographic characteristics of study population.

Parameter		n (%)/Mean (±)
Women		10 (33%)
Age of respondents		20.3 ( ± 2.5)
No of years of education		15 ( ± 1.5)
Smoking/tobacco use (male n = 20)		15 (75%)
Smoking/tobacco use (female n = 10)		5 (50%)
No of smokers in immediate family	0	7 (23%)
	1	11 (37%)
	2	10 (33%)
	3	2 (7%)
Father smokes	Yes	16 (53%)
	No	14 (47%)
Friends smoke	All of them	4 (13%)
	Most of them	11 (37%)
	Some of them	15 (50%)

**Table 2.** Responses given by young adults about how they perceived tobacco addiction and quitting

Perception	Spectrum of Responses of Young Adults
Tobacco is bad for health, I don’t like it and I don’t smoke	I am not curious, I know about the effects in our body, what happens to our lungs…. it gradually destroys the lungs, (causes) breathing problems, I know very much because my grandmother had a lung infection…. she used to take the sweet tobacco…and she died (19, female, non-smoker)
I am not an addict	smoking for me is not necessary, but I often do that…my headache stops when I smoke (female, 18, trying to quit)
I am an addict, but I can’t stop myself	smoking is bad for health, but for me, smoking is an escape…now it has become an addiction (male, 20, smoker)
I am trying to quit	Nothing is helping me. Actually, you just need a determination that it will have to stop because I have my family and they will not like it if I start having this problem (addiction) with myself (male, 18, trying to quit tobacco and other addictions)
It is not possible to quit	They tried to quit but when they see other people smoking they also start…smoking is an addiction…I tried to quit smoking, I tried hard but now I am smoking again (19, male, smoker)

**Table 3.** Juxtaposition of findings from the qualitative study alongside the policy analysis.

Qualitative findings		Policy analysis	
**Themes**	**Sub-themes**	**Policy addresses**	**Policy gaps**
Tobacco initiation	Curiosity about tobacco and easy access to tobacco products	1. Sale under 18 is legally restricted. 2. Tobacco is legally banned from being sold near educational institutes. 3. Regular Anti-Tobacco message in movies and other media.	1. Sale of unpackaged/loose tobacco is not restricted. 2. Taxation does not make the price unaffordable by the youth and others. 3. Sale near colleges, hospitals is freely done till date. 4. Producing ID for age is not mandatory while buying tobacco.
	Cultural beliefs: ‘Smoking in moderation is fine’	1. Anti-tobacco campaigns targeted at heavy smokers, e.g. father coughing in a smoke-filled room in presence of daughter.	1. Implementation of banning smoking in public places is not universally implemented or monitored by civil authorities.
	Knowledge on ill effects of tobacco is not shared adequately: ‘not all who smokes gets cancer’	1. Awareness exists as part of national campaigns being shown on television, movies and other mass media.	1. Policy does not mandate awareness campaigns for youth. 2. Education about tobacco isn’t mandated in school levels or in any curriculum based study. 3. The objective effectiveness of national campaigns unknown.
	Peer influences: ‘All my friends smoke’	1. Policy does not address socio-cultural and behavioural aspects of smoking directly.	1. Policy does not address controlling of passive smoking hazards. 2. Policy does not specifically utilise the full potential of positive peer influences in young people
Maintenance of tobacco use	Denial in youth: ‘I am not an addict’	1. Awareness and educational campaigns targeting young population by using celebrity role models (Cricketers, players, movie stars, etc.).	1. Not enough measures to restrict tobacco use near educational institutions. 2. The effects of youth education and the penetration of such mass media campaigns are not widely known in literature.
	Being fatalistic ‘Smokers will find a way’	1. Package warnings, penalty for smoking in public places, advertisement ban all targeted towards smoking cessation.	1. Not enough restrictive measures exist in the community and the cessation is left on the prerogative of individual smokers. 2. Public smoking penalties need to be adequate to be a deterrent; taxation and other measures need to ensure tobacco is not affordable by a large section of the society.
	Failure to quit in spite of the wish to quit.	1. Cessation (over the counter) drugs available. Cessation clinics as part of govt. establishments available.	1. Very few services that directly targets and assists young people with tobacco cessation in government or private sector. 2. Youth not aware of cessation support. General practitioners and family medicine practitioners in community not adequately sensitised on tobacco counselling and advice.
Anti-tobacco measures	Sale of tobacco	1. Legislation provides a framework on sale of tobacco.	1. Sale to minors is widely practiced.
	Taxation	1. Legislation addresses taxation as one of the controlling measures.	1. Legislation does not address adequate taxation and as a result, tobacco is sold cheaply in India.
	‘Proper packaging of tobacco products may work for some smokers’	1. Package warning widely implemented in most of the tobacco items.	1. Misleading labels as ‘light’, ‘ultra-mild’ gives a false impression of lesser damage. The effects of packaging on youth and other users are not widely available in literature. 2. Package warning is futile in case of selling loose cigarettes, which is very common in youth.
